# A Prominent Role for DC-SIGN^+^ Dendritic Cells in Initiation and Dissemination of Measles Virus Infection in Non-Human Primates

**DOI:** 10.1371/journal.pone.0049573

**Published:** 2012-12-05

**Authors:** Annelies W. Mesman, Rory D. de Vries, Stephen McQuaid, W. Paul Duprex, Rik L. de Swart, Teunis B. H. Geijtenbeek

**Affiliations:** 1 Department of Experimental Immunology, Academic Medical Center, University of Amsterdam, Amsterdam, The Netherlands; 2 Viroscience lab, Erasmus MC, Rotterdam, The Netherlands; 3 Tissue Pathology, Belfast Health & Social Care Trust, Queen’s University of Belfast, Belfast, United Kingdom; 4 Department of Microbiology, Boston University School of Medicine, Boston, Massachusetts, United States of America; Institut Pasteur, France

## Abstract

Measles virus (MV) is a highly contagious virus that is transmitted by aerosols. During systemic infection, CD150^+^ T and B lymphocytes in blood and lymphoid tissues are the main cells infected by pathogenic MV. However, it is unclear which cell types are the primary targets for MV in the lungs and how the virus reaches the lymphoid tissues. *In vitro* studies have shown that dendritic cell (DC) C-type lectin DC-SIGN captures MV, leading to infection of DCs as well as transmission to lymphocytes. However, evidence of DC-SIGN-mediated transmission *in vivo* has not been established. Here we identified DC-SIGN^hi^ DCs as first target cells *in vivo* and demonstrate that macaque DC-SIGN functions as an attachment receptor for MV. Notably, DC-SIGN^hi^ cells from macaque broncho-alveolar lavage and lymph nodes transmit MV to B lymphocytes, providing *in vivo* support for an important role for DCs in both initiation and dissemination of MV infection.

## Introduction

Measles virus (MV) is a highly contagious virus, transmitted via the respiratory route. Despite the availability of a safe and effective vaccine and increased vaccination coverage, measles outbreaks are still associated with high levels of childhood mortality [1]. Clinical symptoms appear approximately two weeks after MV infection and are associated with a profound immune suppression that lasts for several weeks to months and leads to enhanced susceptibility to opportunistic infections [2], [3].

The entry receptor for wild-type MV is CD150 (signaling lymphocyte activation molecule or SLAM) [4], expressed mainly by subsets of B and T lymphocytes and dendritic cells (DCs). Recently, Poliovirus-receptor-like-4 (PVRL4 or Nectin-4) was identified as the epithelial entry receptor. This protein is exclusively expressed on the basolateral side of epithelial cells and usage of this receptor is associated with late stages of disease progression and host-host transmission [5], [6]. Macaque infection studies demonstrated that MV is detected at the peak of infection in the lungs, peripheral blood mononuclear cells (PBMC) and all lymphoid tissues [7]–[9]. We have shown in *in vivo* studies using a pathogenic recombinant (r)MV expressing enhanced green fluorescent protein (EGFP) that memory CD150^+^ B and T lymphocytes are the predominant cells infected in blood and lymph nodes during the peak of infection [10], [11]. However, it remains unclear which cells are the first target cells after aerosol infection and how the virus is disseminated from lungs to the lymphoid tissues.

Dendritic cells (DCs) have been suggested to play an important role in virus transmission. DCs are professional antigen presenting cells that migrate to lymph nodes upon encountering pathogens and induce a pathogen-specific immune response [12]. Besides playing a pivotal role in shaping the immune response to MV [3], [13]–[16], DCs have also been suggested to transmit MV to lymphocytes [13]. Several *in vitro* studies have shown that DCs efficiently transmit viruses such as HIV-1 and MV to lymphocytes [13], [17], but little is known about virus transmission *in vivo*.

The C-type lectin Dendritic Cell-Specific Intercellular adhesion molecule-3-Grabbing Non-integrin (DC-SIGN) is an attachment receptor for MV [13], [18]. DC-SIGN has a high affinity for mannose containing structures, including glycosylated viral proteins such as human immunodeficiency virus (HIV) type 1 gp120 [17], [19] and the MV fusion (F) and hemagglutinin (H) transmembrane glycoproteins [13]. *In vitro* models demonstrate that interaction of MV with human DC-SIGN enhances DC infection as well as transmission of MV from DCs to both CD4^+^ and CD8^+^ T cells. MV transmission can occur independent of DC infection (in *trans*) through capture of the virus and transmission to target cells [18]. Due to the widespread distribution of DC-SIGN^+^ DCs throughout the respiratory tract [18] and their capacity for viral transmission, DCs have been suggested as key players in initiating MV infection of the host and disseminating the virus from the first site of infection to local or draining lymphoid tissues. However, it is not known whether DC-SIGN is involved in virus transmission *in vivo*.

We recently studied the early events after MV infection of macaques with the pathogenic rMV^KS^EGFP strain via aerosol inhalation. EGFP-positive cells were exclusively detected in the alveolar lumen or attached to the alveolar epithelium of the lungs 2 days post-infection (d.p.i.). From 3 d.p.i. clusters of MV-infected cells were detected in bronchus-associated lymphoid tissue (BALT) and in the tracheo-bronchial lymph nodes (TBLN) [20]. The initial target cells morphologically resembled either DCs or alveolar macrophages (AM), but their identity and role in viral transmission remained unknown. In order to gain more understanding of the *in vivo* function of DCs in measles, we here investigated the phenotype of the first target cells and their function in the early stages of MV infection.

After aerosol infection with the pathogenic rMV^KS^EGFP strain, we observed that DC-SIGN^hi^ cells in the lungs and lung-draining lymph nodes of non-human primates were among the first MV-infected cells. *Ex vivo* cultured lung tissue from infected animals showed focal infection that spread outward during culture and after 8 days most infected cells were T lymphocytes, suggesting that DC-SIGN^hi^ cells in lungs are a first target and transmit the virus to lymphocytes after initial infection. Furthermore, isolated DC-SIGN^hi^ DCs interacted with MV and were able to transmit the virus to lymphocytes more efficiently than DC-SIGN^-^ cells. Our data strongly suggest an important role for DC-SIGN in dissemination of and infection with measles virus *in vivo*.

## Results

### DC-SIGN^+^ Cells are Early Target Cells in Lungs and Tracheo-bronchial Lymph Nodes

**Figure 1:**
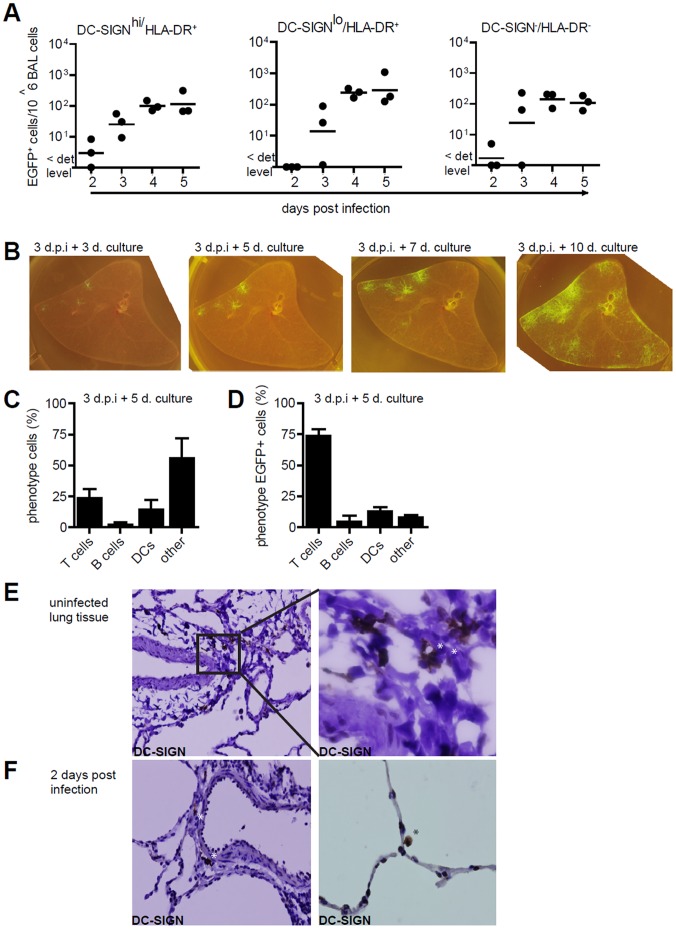
Infection of DC-SIGN^+^ cells in the lung. (A) Infection of the DC-SIGN^hi^, DC-SIGN^lo^ and DC-SIGN^-^ cells in BAL from MV-infected macaques, determined by detection of EGFP in flow cytometry at day 2–5 d.p.i. Each dot represents an individual animal. Lines indicate geometric means. (B) Macroscopic images from EGFP^+^ lung slices collected 3 d.p.i., cultured for additional 3,5,7 or 10 days. (C) Phenotype of cells migrating from the *ex vivo* cultured lung slice, collected from supernatant after 5 days of culturing (D) Phenotype of EGFP^+^ cells collected from lung slice medium. (E-F) DC-SIGN expression on lung sections from uninfected macaques (E) or 2.d.p.i. (F) Asterisks indicate DC-SIGN reactivity.

Cynomolgus macaques were infected with a high dose (10^6^ TCID_50_) of rMV^KS^EGFP by aerosol inhalation [20]. To investigate the role of DCs during early MV infection, we analyzed DC-SIGN, HLA-DR and EGFP expression of broncho-alveolar lavage (BAL) cells collected 2–5 d.p.i. The size of the DC-SIGN^hi^/HLA-DR^+^ and DC-SIGN^lo^/HLA-DR^+^ cell subpopulations in the total BAL population remained stable and relative numbers in the total population at 4 d.p.i were 16.1% ±4.5 and 18.8% ±4.5, respectively. These subpopulations represented the antigen presenting cells, whereas the DC-SIGN^−/^HLA-DR^-^ cells included the lymphocytes. We were unable to detect any macroscopic fluorescence at day 2 d.p.i. [20] and therefore measured infection by EGFP using flow cytometry, the number of MV-infected cells detected was in the same range as previously reported [20]. EGFP^+^ DC-SIGN^hi^ cells were detected in 2/3 animals at the earliest time point, 2 d.p.i., whereas no EGFP^+^ DC-SIGN^lo^/HLA-DR^+^ cells were present on day 2 (Figure 1A). In 1/3 animals, few (<10/10^6^ BAL cells) DC-SIGN^−/^HLA-DR^-^ infected cells were identified in BAL on 2 d.p.i. Low numbers of MV-infected cells and low levels of replication 2 d.p.i. did not allow for detection of MV-captured Ag independent of infection on DCs. These data indicate that the DC-SIGN^+^ cell population is among the first to become infected by MV. From 3 d.p.i. onwards, EGFP^+^ DC-SIGN^hi^ cells were detected in all animals (n = 3 per time point) and the number of MV-infected cells increased over time. Similarly, the infection of DC-SIGN^lo^/HLA-DR^+^ as well as DC-SIGN^−/^HLA-DR^-^ cells increased over time, suggesting that the virus was disseminated to these populations.

At 3 d.p.i. live agarose-inflated lung slices from 2 animals were cultured to visualize infection over time (Figure 1B). Small areas of EGFP fluorescence were visible after culture for 3 days demonstrating MV infection of single cells that spread throughout the tissue, since a clear focal spread of MV was observed the next 7 days. After 5 days of culture, cells emigrating from the tissue into the supernatant were analyzed for immune cell markers to determine the phenotype of MV-infected cells. The total population consisted mainly of cells negative for lymphocyte or DC markers (Figure 1C). In contrast, more than 70% of the EGFP^+^ population cells were CD3^+^ (T lymphocytes), whereas EGFP^+^ DC-SIGN^+^/HLA-DR^+^ (DCs) and CD20^+^ cells (B lymphocytes) were also identified (Figure 1D). Together, these data suggest that DC-SIGN^+^ cells in the lungs are a target for MV at the earliest time points of infection. At later time points DC-SIGN^−/^HLA-DR^-^ cells (mainly T-lymphocytes) became the predominant MV-infected cell-population.

We next analyzed the localization of DC-SIGN^+^ cells in the lungs. In lung tissue from both uninfected and 2 d.p.i, DC-SIGN expression was mainly detected on cells with irregular, DC-like morphology located in close proximity of the lumen and large round cells lining the alveolar lumen (Figure 1E and F), suggesting that DC-SIGN^hi^ cells can encounter inhaled viral particles, explaining their infection at the earliest time point.

**Figure 2:**
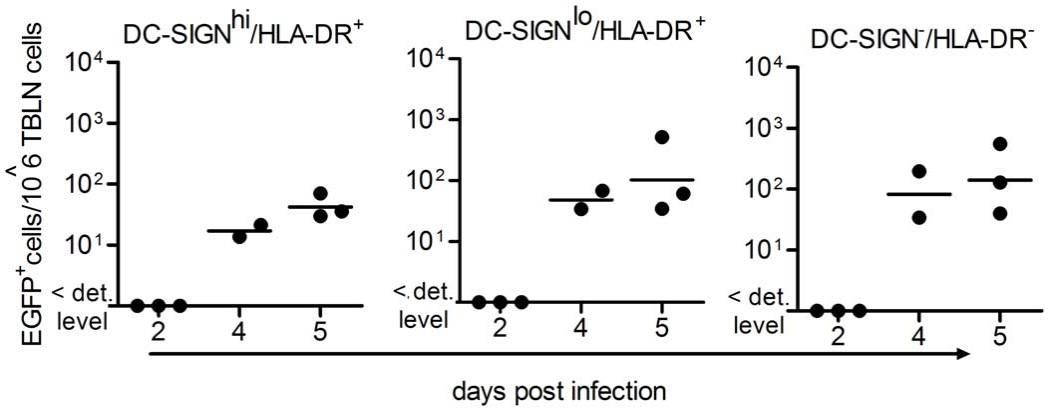
Infection in the tracheo-bronchial lymph nodes. Infection of the DC-SIGN^hi^, DC-SIGN^lo^ and DC-SIGN^-^ cells in TBLN from MV-infected macaques, determined by detection of EGFP in flow cytometry from 2–5 d.p.i. Each dot represents an individual animal. Lines indicate geometric means.

Next we investigated the appearance of infected cells in the lung draining TBLNs. Single cell suspensions of TBLNs collected 2, 4 or 5 d.p.i. were analyzed for EGFP, HLA-DR and DC-SIGN expression (Figure 2). We could not detect any MV-infected cells in TBLN 2 d.p.i. MV-infected DC-SIGN^hi^/HLA-DR^+^ cells, DC-SIGN^lo^/HLA-DR^+^ cells as well as DC-SIGN^−/^HLA-DR^-^ cells were detected 4 d.p.i. and the number of infected cells increased at day 5 in all three subsets. These data suggest that DC-SIGN^hi^HLA-DR^+^ cells are an initial target for MV in the lung at day 2 and dissemination from lung to draining TBLN occurs after day 2 and results in infection of antigen-presenting cell and lymphocyte populations.

### Phenotype of DC-SIGN^+^ Cells in Macaques

**Figure 3:**
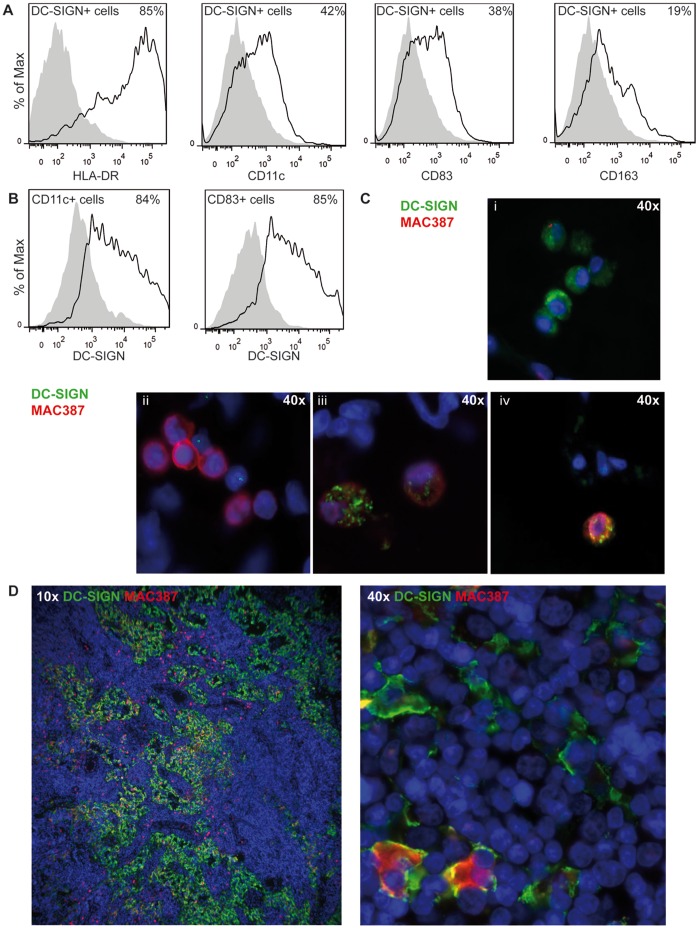
DC-SIGN is expressed by DCs and a subset of macrophages in lymph nodes. (A) TBLN cells were stained for DC-SIGN in combination with DC and macrophage markers and analyzed by flow cytometry. Gray areas show negative controls (DC-SIGN single staining). Percentages of positive cells expressing the markers are annotated in the upper right corner. (B) DC-SIGN expression of CD11c^+^ and CD83^+^ cells in TBLNs. (C-D) Dual immunofluorescence staining of DC-SIGN (green) and MAC387 (red) in lung sections 2 or 3 d.p.i. (C i-iv) and axillary lymphoid tissue 4 d.p.i. (D). Nuclei are stained blue with Hoechst.

In order to study the role of DC-SIGN^+^ cells in lymphoid tissue in macaques, we characterized these cells by flow cytometry and immunofluorescence microscopy. Cells isolated from TBLNs of uninfected animals were stained for DC-SIGN and multiple monocyte markers: HLA-DR, DC-marker CD11c, DC maturation marker CD83, and macrophage scavenger receptor CD163. All DC-SIGN^+^ cells expressed HLA-DR, whereas CD11c and CD83 were only expressed by part of the DC-SIGN^+^ population (Figure 3A). However, DC-SIGN was expressed by almost all CD83^+^ and CD11c^+^ cells (Figure 3B). In addition, we identified a small subset of CD163^+^ cells in the TBLN that was partially positive for DC-SIGN (Figure 3A). These data suggest that DC-SIGN is expressed by all CD11c^+^ and CD83^+^ DCs as well as a subset of macrophages in macaques.

Next we analyzed distribution of DC-SIGN^+^ DCs and macrophage marker MAC387^+^ macrophages in both lungs and draining lymph nodes from infected macaques. Because EGFP-expressing rMV was used, unstained infected sections served as control besides isotype controls, and gave no signal in the EGFP channel. In lung sections collected 2 or 3 d.p.i. almost no co-localization was observed between DC-SIGN and MAC387. Most large cells near the alveolar lumen were either DC-SIGN^+^ (Figure 3Ci) or MAC387^+^ (Figure 3Cii). Some co-localization as seen in TBLN was observed (Figure 3Ciii and iv). In addition, many DC-SIGN^+^ cells were observed in peripheral lymphoid tissues, including axillary lymph nodes (Figure 3D) and tonsils (data not shown), at 4–5 d.p.i. the time at which infection just reached a systemic phase [20]. Thus it seems that infection of lymphoid tissue is associated with abundant numbers of DC-SIGN^+^ cells in lymphoid tissues. In the axillary lymph node 4 d.p.i., the expression patterns of DC-SIGN and MAC387 indicated two distinct cell populations, although co-localization was observed in a relatively small number of cells (Figure 3D). These data show that DC-SIGN is expressed predominantly by DCs and some macrophages in the lungs and lymph nodes in uninfected and MV-infected animals.

### Macaque DC-SIGN Binds MV and Transmits the Virus to Target Cells

**Figure 4:**
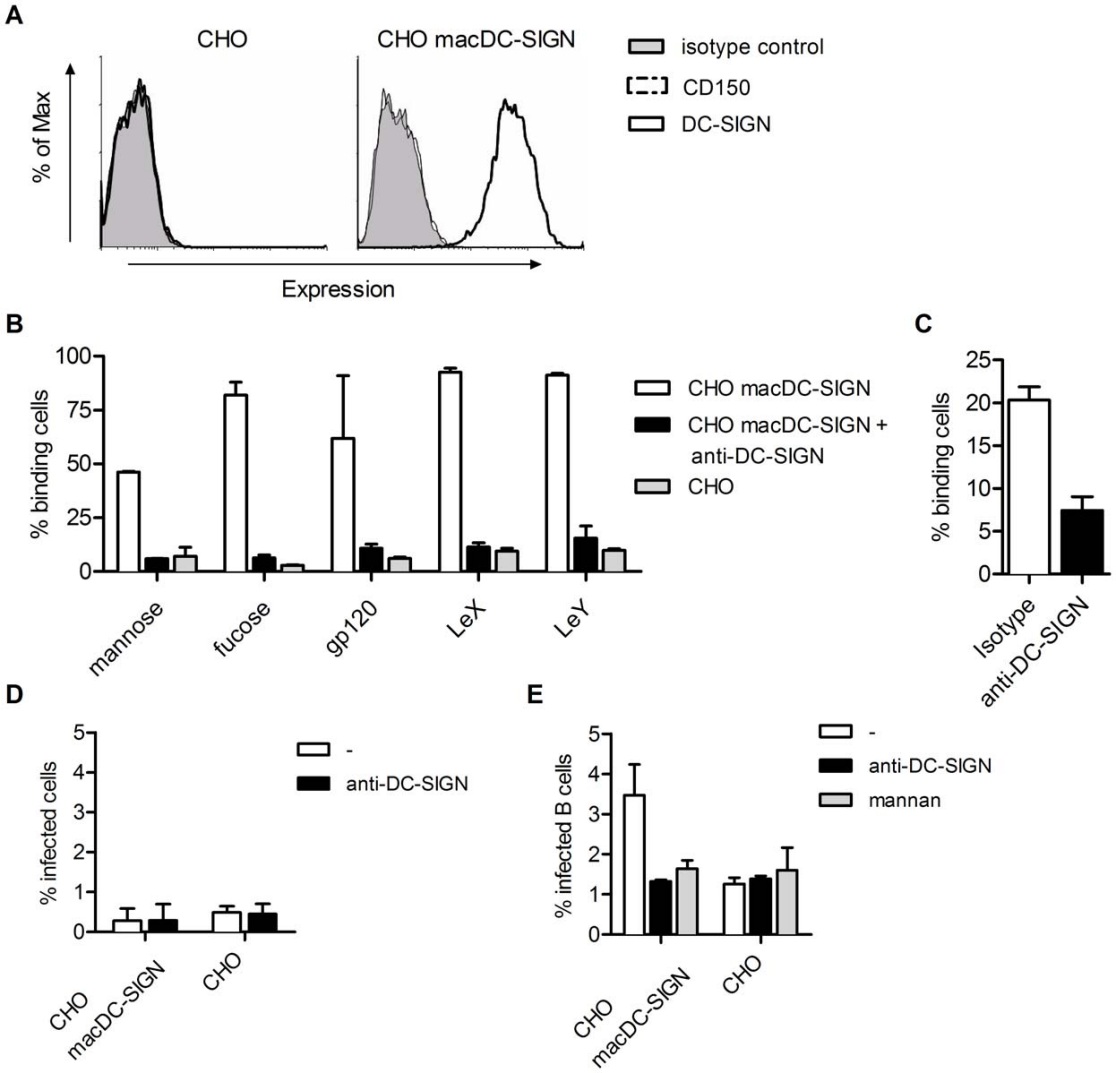
Macaque DC-SIGN binds mannose structures including MV and transmits MV to CD150^+^ target cells. (A) CHO cells were transfected with macDC-SIGN. The mean expression levels of DC-SIGN and CD150 of the parental cell line and transfectants are depicted. Gray areas represent isotype controls. (B) MacDC-SIGN binds mannose and fucose structures on fluorescent beads. Binding was blocked by anti-DC-SIGN (20 ug/ml. (C) CHO transfectants were pre-incubated with anti-DC-SIGN antibodies or isotype control (20 ug/ml) before incubation with FITC-labeled UV-inactivated MV. Binding was measured by flow cytometry. (D) Parental and transfected CHO cells were infected with rMV^KS^EGFP (MOI 1) and 48 hours post infection EGFP levels were measured in FACS. (E) Cells were incubated with rMV^KS^EGFP (MOI 3). After 3 hours cells were washed and CD150^+^ Raji cells were added to the culture. Infection of Raji cells was determined by measuring EGFP in flow cytometry. Transmission was blocked by pre-incubating CHO macDC-SIGN cells with anti-DC-SIGN (20 ug/ml) or mannan (0.25 mg/ml). All data are representatives for at least 2 independent experiments. Error bars represent standard deviations of duplicates.

Human DC-SIGN is an attachment receptor for MV and mediates both infection of DCs (via CD150) and transmission to target cells (via CD150 or PVRL4), which can be independent of infection of DCs [13], [18]. Non-human primate homologues are ≥94% identical to human DC-SIGN and share a high affinity for ICAM-2, ICAM-3 and cross-react with multiple monoclonal antibodies against human DC-SIGN [21]. We investigated whether macDC-SIGN binds MV and is able to transmit the virus to target cells. Chinese hamster ovary (CHO) cells transfected with macDC-SIGN expressed high levels of DC-SIGN, but were negative for MV entry receptor CD150 (Figure 4A). Next we investigated the interaction of macDC-SIGN with different mannose- and fucose-containing structures using the fluorescent bead binding assay [17]. CHO cells expressing macDC-SIGN efficiently interacted with mannose, fucose and fucose-containing structures such as Lewis X and Lewis Y (Figure 4B). Furthermore, macDC-SIGN-expressing CHO cells interacted strongly with the HIV-1 envelope glycoprotein gp120. The interaction was specific for macDC-SIGN since antibodies against DC-SIGN blocked the binding to background levels comparable to the parental cell-line (Figure 4B). To investigate interaction of macDC-SIGN with MV, CHO cells expressing macDC-SIGN cells were incubated with FITC-labeled MV. MacDC-SIGN^+^ CHO cells bound MV as determined by flow cytometry (Figure 4C). Binding to fluorescent virus particles was macDC-SIGN-dependent, since it was inhibited by a blocking antibody against DC-SIGN compared to the isotype control. Incubation of CHO and CHO-macDC-SIGN cells with rMV^KS^EGFP for 48 hours did not lead to infection, showing that macDC-SIGN does not facilitate MV entry (Figure 4D). To investigate the role of macDC-SIGN in MV transmission, cells were incubated with rMV^KS^EGFP, washed and subsequently co-cultured with CD150^+^ Raji B cells for 24 hours. CHO cells expressing macDC-SIGN transmitted MV to the B cells. Pre-treating cells with mannan or DC-SIGN blocking antibodies decreased transmission to background levels (Figure 4E). Thus, macDC-SIGN interaction with MV mediates viral binding and transmission of the virus to lymphocytes independent of infection.

### DC-SIGN^+^ Cells from Both BAL and Lymph Nodes Transmit MV to Lymphocytes

**Figure 5:**
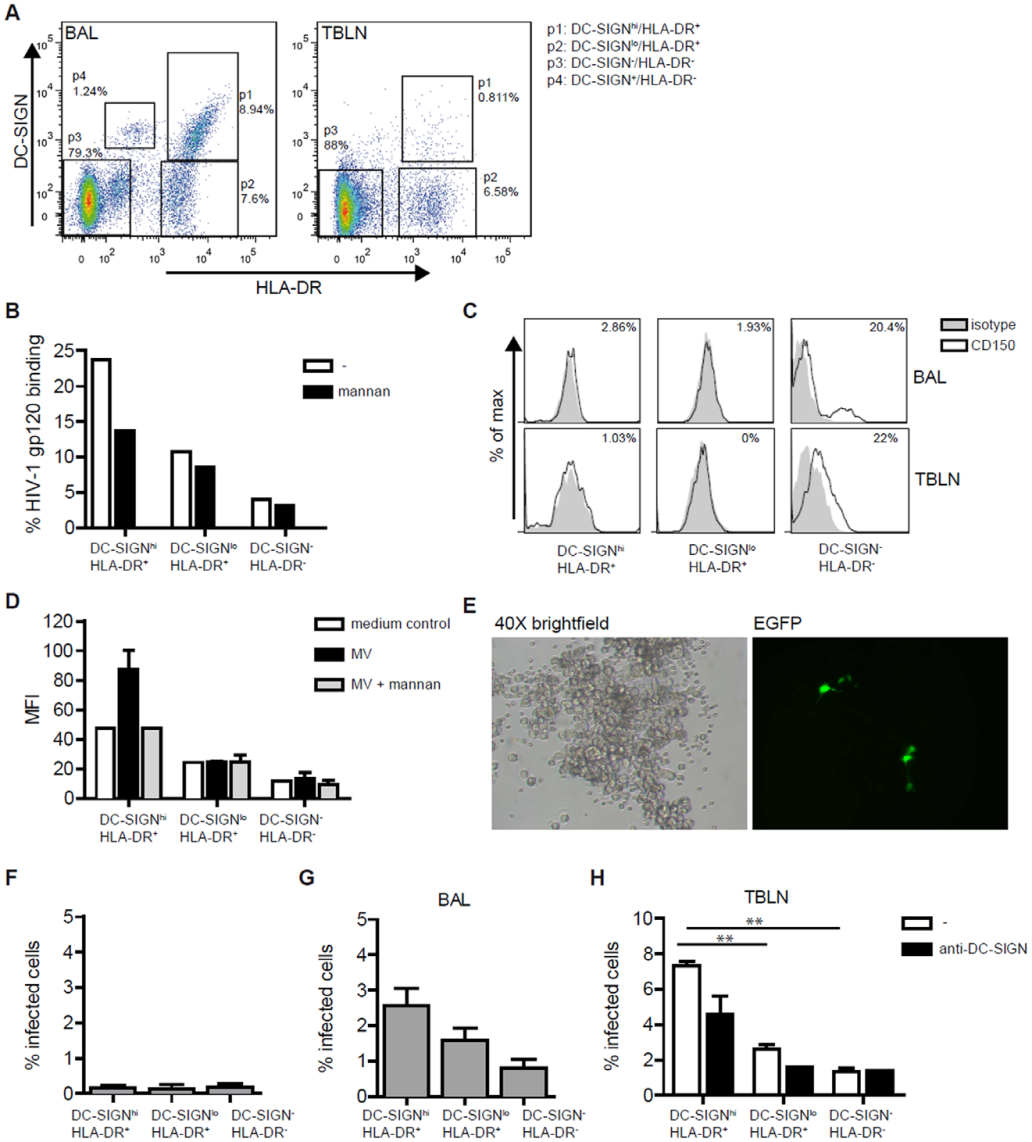
DC-SIGN expressed by BAL and lymph node cells enhances MV transmission. (A) DC-SIGN^hi^/HLA-DR^+^ (p1), DC-SIGN^lo^/HLA-DR^+^ (p2) and HLA-DR^−/^DC-SIGN^-^ (p3) cells were sorted by FACS from BAL and lymphoid tissues of uninfected animals. Gates and percentages of the subsets are depicted. A representative FACS plot of 3 independent experiments is shown. (B) DC-SIGN on sorted BAL cells binds HIV-1 gp120-coated fluorescent beads. Binding was blocked by mannan (0.25 mg/ml). Representative data of 2 independent experiments are shown. (C) CD150 expression of the 3 subsets from BAL and TBLN (black lines) compared to isotype controls (gray areas). Percentages of CD150^+^ cells are depicted in the histograms. (D) Sorted cells from lymph nodes of an uninfected animal were incubated with fluorescently labeled rMV^KS^EGFP or medium control. Binding was measured by flow cytometry and the mean fluorescent intensity (MFI) is depicted. (E) Representative example of 2 indepedent *ex vivo* infections of BAL cells with rMV^KS^EGFP (MOI 3) 24 hours post infection. The left (brightfield) and right (EGFP fluorescent) panel are corresponding pictures. (F) Infection of sorted DC-SIGN subsets with rMV^KS^EGFP (MOI 3) was determined by measuring EGFP in FACS. Means and standard deviations of 2 independent experiments are shown. (G-H) Cells were incubated with rMV^KS^EGFP (MOI 1) for 3 hours. Then the cells were washed and B cells were added. After 24 hours EGFP expression was measured by flow cytometry. For BAL cells, combined data of 2 independent experiments are shown (G). Cells isolated from lymph nodes were pre-incubated with blocking anti-DC-SIGN antibodies (20 ug/ml) for 30 minutes. ** p<0.01 (H). Bars represent the mean of duplicates.

Next we investigated whether DC-SIGN^+^ cells of macaques transmit MV to lymphocytes *ex vivo*. DC-SIGN^hi^/HLA-DR^+^, DC-SIGN^lo^/HLA-DR^+^ and DC-SIGN^−/^HLA-DR^-^ cells were purified by flow cytometry sorting from BAL and lymphoid tissues of uninfected macaques (Figure 5A). In addition to these 3 populations, we detected a small DC-SIGN^+^/HLA-DR^-^ in BAL (designated as p4 in Figure 5A). However this population consisted of too few cells for additional phenotyping. Expression of DC-SIGN following sorting was confirmed at both the protein and mRNA level (data not shown). Functionality of DC-SIGN expressed by the sorted cells from BAL was investigated with the bead binding assay (Figure 5B). DC-SIGN^hi^ cells interacted with HIV-1 gp120, which was inhibited by mannan. The other subsets interacted much less with HIV-1 gp120. Similar results were obtained with cells from TBLN (not shown), supporting a role for macDC-SIGN^+^ cells in virus capture.

CD150 was expressed by a subpopulation of DC-SIGN^−/^HLA-DR^-^ cells, which included T and B lymphocytes, in BAL. Low CD150 expression was detected on most cells in TBLN. DC-SIGN^hi^/HLA-DR^+^ and DC-SIGN^lo^/HLA-DR^+^ cells expressed very low levels of CD150 in BAL and the receptor was not detected on the cell-surface on DC-SIGN^lo^/HLA-DR^+^ cells in TBLN (Figure 5C). These data suggest that the HLA-DR^+^ cells in the lungs are not highly susceptible to MV infection without activation. However, we showed that DC-SIGN is able to capture MV for transmission. Therefore, we investigated the interaction of the different cells with fluorescently labeled MV. Notably, DC-SIGN^hi^ cells isolated from lymphoid tissue bound more efficiently to fluorescently-labeled MV and the binding was blocked to background levels with the inhibitor mannan. In contrast, DC-SIGN^lo^ and DC-SIGN^-^ cells did not bind MV (Figure 5D). These data strongly suggest that despite low expression of CD150, the DC-SIGN^hi^ cells in contrast to DC-SIGN^lo^ and DC-SIGN^-^ cells efficiently capture MV.

We subsequently investigated the transmission of MV to Raji B cells by the different subsets isolated from uninfected macaques. *Ex vivo* infection of unsorted BAL cells was inefficient and only 1% EGFP^+^ cells was detected 24 hours after infection with rMV^KS^EGFP (MOI 3). However, microscopic analysis showed that a number of infected cells in the total BAL cell population showed a DC-like morphology (Figure 5E). *Ex vivo* infection levels of the sorted DC-SIGN/HLA-DR subsets were below 0.5% 24 h.p.i., and no differences were observed between subsets (Figure 5F). Inefficient infection of *ex vivo* sorted cell populations might indicate that lung factors facilitate infection *in vivo*. Thus, transmission by the sorted cells to B cells was primarily due to infection independent of direct infection of the subsets. The different purified cell populations from BAL were pulsed with rMV^KS^EGFP for three hours, and after washing CD150^+^ Raji cells or B-LCL cells were added. Infection of the lymphocytes was measured in the co-culture at day 1 or 2 by flow cytometry. Notably, we observed a higher transmission of MV to target lymphocytes by the DC-SIGN^hi^ cells compared to both DC-SIGN^lo^/HLA-DR^+^ and DC-SIGN^−/^HLA-DR^-^ subsets (Figure 5G). Similarly, transmission of MV by DC-SIGN^+^ subsets isolated from TBLNs was significantly higher compared to DC-SIGN^lo^/HLA-DR^+^ and DC-SIGN^−/^HLA-DR^-^ subsets. Transmission of MV by DC-SIGN^hi^/HLA-DR^+^ cells was partially dependent on DC-SIGN, since it was inhibited by blocking antibodies against DC-SIGN (Figure 5H). These data suggest that DC-SIGN^+^ cells capture MV and facilitate infection of lymphocytes, thereby amplifying local and systemic spread.

## Discussion

In this study we have investigated the role of DC-SIGN^+^ DCs during the early stage of MV infection using both *in vivo* and *ex vivo* models. After aerosol infection of macaques with the pathogenic rMV^KS^EGFP strain, we observed that DC-SIGN^hi^ DCs were among the first infected cells in the lungs of macaques. These cells were sorted by flow cytometry and DC-SIGN^hi^ DCs were efficient in MV capture as well as MV transmission to lymphocytes. Furthermore, using cell-lines expressing macDC-SIGN we showed that macDC-SIGN is an attachment receptor for MV, similar to human DC-SIGN. This suggests that DC-SIGN^+^ DCs are an initial target for MV for viral transmission and thereby contribute to viral dissemination upon aerosol infection. Notably, although we detected MV-infected DC-SIGN^hi^ cells at the earliest time point in the lungs, DC-SIGN^hi^ DCs as well as macDC-SIGN-transfected cells were able to transmit MV independent of infection. Thus, transmission of MV to lymph nodes could depend on infected as well as non-infected DC-SIGN^+^ cells in the lungs.

Epithelial cells in the upper respiratory tract have long been considered as early target cells in MV infection [2]. Epithelial cells express the recently identified entry receptor PVRL4 exclusively on the basolateral side of the cells, and no CD150, and therefore cannot play a role in initiation of infection [5], [6]. DCs and AMs have been proposed as initial target cells for the virus since these cells express CD150 [18], [20], [22]. Non-human primate infection studies have shown that large mononuclear cells in the lung are early targets for MV after aerosol infection [20]. The identity of these cells was not determined but it was suggested that these cells disseminate MV infection from lungs to BALT and subsequently to TBLNs draining the lungs [20]. Moreover, Ferreira *et al*, using a transgenic mouse model after both intranasal and intraperitoneal infection, observed MV infection of AMs and DCs in the airways preceding infection in mediastinal lymph nodes [22]. Here, we observed MV-infected DC-SIGN^hi^/HLA-DR^+^ cells 2 d.p.i., whereas no infected DC-SIGN^lo^/HLA-DR^+^ cells were identified at that time point. DC-SIGN^+^ cells in the lungs were often located in or adjacent to the lumen, where virus is encountered. The cells were positive for CD11c, demonstrating that these cells are myeloid and not plasmacytoid DCs. Therefore, DC-SIGN^+^ DCs in macaque lungs could be a target for MV and play a role in dissemination.

Furthermore, we observed the infection in the lungs spread throughout foci, where mainly T cells were affected. Since DCs are important for inducing BALT formation in response to viral infections and for maintenance of the organized BALT structure [23], [24], DC-SIGN^+^ DCs might spread initial infection to BALT and enhance local MV replication in the lungs, as well as activate immune cells in BALT. A role for DCs in amplification of MV infection in the lungs is supported by a previous finding where MV-infected CD11c^+^ cells were observed in small infected foci in the lungs from 3 d.p.i. [20]. In addition, CD11c^+^ DCs have been identified as major target cells in peripheral tissues and were often observed in conjunction with infected T lymphocytes, suggesting viral transmission between the cells [10]. We observed from 3 d.p.i. that the infection in all animals spread to other cells including the DC-SIGN^−/^HLA-DR^-^ cells, which contain activated CD150^+^ lymphocytes. Furthermore, *ex vivo* lung cultures showed that these cells become the predominant infected population in lungs 8 d.p.i. Notably, although we measured infection *in vivo*, we detected low levels of CD150 on DC-SIGN^hi^ cells from BAL and lymph nodes. This is consistent with previous findings, where low levels of CD150 in the respiratory tract are detected [18]. This could indicate that high levels of DC-SIGN allow infection when CD150 expression is low, since DC-SIGN enhances MV infection [13].

Previously, DC-SIGN was detected on cells expressing CD11c, described as a marker for myeloid DCs and a subset of AMs in the human lung [25], [26] and in macaques no co-localization of MAC387 and CD11c in BALT cells 3 d.p.i. was seen [20]. Here, we observed that DC-SIGN was predominantly expressed by CD11c^+^ DCs, whereas a subpopulation of MAC387^+^ macrophages expressed DC-SIGN. This subpopulation might represent AMs since it has been described that AMs express DC-SIGN after activation [27], [28]. Pulmonary DCs are professional antigen presenting cells and equipped to rapidly migrate to the draining lymph node upon encountering virus in the lung [29]–[31]. Besides their role as migratory antigen presenting cells, DCs also play a major role in MV pathogenesis by contributing to infection and immune suppression [2]. DC-SIGN contributes to these processes since interactions with viral pathogens enhance infection and viral uptake as well as modulate immune responses [14], [17], [18]. Furthermore, MV can induce TLR2 activation [32], which might also be involved in DC-mediated immune responses as well as induction of migration from lungs to LN. In contrast, AMs are crucial for maintaining homeostasis in steady-state condition and restoring homeostasis after infection [33]. This classical division of DCs as migrating antigen presenting cells and macrophages as tissue residents further supports a role for DCs transferring viruses to lymphoid cells [17], [18].

Our data show that at day 2 d.p.i. no MV infection was observed in draining lymph nodes, whereas we detected MV-infected DC-SIGN^hi^ DCs in the lungs. At 4 d.p.i. we observed MV infection of different cell types in draining lymph nodes, suggesting that the virus had spread from lung to lymph nodes. In our previous study, MV-infected cells with the morphology of DCs were detected migrating through the endothelium early in infection [20], suggesting that infected or MV-bound DCs migrate from the lungs to TBLNs to transmit the virus to T lymphocytes. Migration of DCs after MV infection is further supported by a strong induction of lymph node homing chemokine CCR7 [34]. In the TBLNs draining the lungs, DC-SIGN was mainly expressed by CD11c^+^ DCs and DC maturation marker CD83^+^ DCs, implying a role for these cells in immune activation.

We and others have shown that DC-SIGN functions as an attachment receptor for viruses such as MV and HIV-1, which can enhance infection thereby promoting transmission. However, infection of DCs is not required for viral DC-SIGN-mediated transmission [13], [17], [18]. Here we showed that macDC-SIGN efficiently captured MV and transmitted it to lymphocytes independent of MV infection. Further, isolated DC-SIGN^hi^ cells from the lungs or TBLNs bound MV and enhanced transmission of the virus to B cells. DC-SIGN antibodies partially blocked transmission, indicating that DC-SIGN was involved, but other factors, such as heparan sulfates [35], [36] might also contribute. B cell infection could be a result of *trans*- or *cis* infection, although inefficient infection of DC-SIGN^hi^ cells supports the model of transmission independent of DC infection in our *ex vivo* assays. We therefore hypothesize that DC-SIGN^+^ cells, infected or uninfected, facilitate transfer to lymphocytes that are the main replicators for MV.

Overall, we have shown a role for DC-SIGN in MV transmission, using a macaque infection model. Our results using primary cells from the lungs and lymphoid tissue are in concordance with the results previously obtained with human monocyte-derived DCs [18]. *In vitro* studies of DC-SIGN-mediated mechanisms however do not take into account the plasticity of the phenotype and function of innate immune cell subsets [37], [38]. Taken together, these data support the idea that MV targets HLA-DR^+^ cells, including DC-SIGN^+^ cells, in the lungs directly after infection. This study provides a better understanding of initiation of MV infection *in vivo*, which may be beneficial for development of an aerosol- or powder-delivered vaccine [39]–[42] to increase immunization coverage.

## Materials and Methods

### Ethics Statement

Animal experiments were conducted in compliance with European guidelines (EU directive on animal testing 86/609/EEC) and Dutch legislation (Experiments on Animals Act, 1997). The protocol was approved by the independent animal experimentation ethical review committee Dier Experimenten Commissie in Driebergen, The Netherlands.

Animals were obtained from the National Institute of Public Health and the Environment, Bilthoven, and transported to the central animal housing facility of the Erasmus MC in Rotterdam. The animals were housed in groups, received standard primate feed and fresh fruit on a daily basis and had access to water ad libitum. In addition, their cages contained several sources of environmental enrichment in the shape of hiding places, hanging ropes, tires and other toys. Animal welfare was observed on a daily basis, animal handling was performed under light anesthesia using ketamine and medetomidine. After handling atipamezole was administered to antagonize the effect of medetomidine.

### Animal Studies

The infection study was previously described by Lemon *et al*. [20]. In short, MV-seronegative cynomolgus macaques (*Macaca fascicularis*) were infected with an estimated dose of 10^5^ CCID50 rMV^KS^EGFP via aerosol inhalation, using a pediatric face mask. Animals were euthanized 2, 3, 4 or 5 d.p.i. (n = 3 per time point). Animals were euthanized by sedation with ketamine (20 mg/kg body weight) followed by exsanguination. For both experiments with infected and uninfected animals, tissues were collected in PBS and directly processed. A broncho alveolar lavage (BAL) was performed post-mortem by direct infusion of 10 ml phosphate buffered saline (PBS) into the right lung lobe. BAL cells were re-suspended in RPMI1640 supplemented with L-glutamine (2 mM), penicillin (100 U/ml), streptomycin (100 mg/ml) and 10% (v/v) heat-inactivated fetal bovine serum (FBS), lymphoid organs were collected during necropsy in PBS for direct preparation of single cell suspensions. Suspensions were obtained by mashing the nodes, using cell strainers with a 100 mm pore size (BD Biosciences) and washing the cells in RPMI1640. Single cell suspensions and BAL cells were counted and used directly for flow cytometry, FACS sorting or *ex vivo* assays.

### Lung Slices

The left lung lobe of 2 animals on 3 d.p.i. was inflated using a solution of 4% (w/v) agarose in PBS mixed 1∶1 with DMEM/Ham’s F12 medium supplemented with L-glutamine (2 mM), 10% (v/v) heat-inactivated FBS, penicillin (100 U/ml) and streptomycin (100 mg/ml). The inflated lung was allowed to solidify on ice and 1 mm slices were cut by hand. Sections were washed in PBS and transferred to a 6-wells plate.

### Antibodies

All antibodies used cross-react with macaques according to the NIH Nonhuman Primate Reagent Resource or the manufacturer. The following antibodies were used for flow cytometry and blocking: DC-SIGN-specific mouse antibodies AZN-D1 and AZN-D2 [17], DCN46 conjugated with PE (BD Pharmingen, San Diego, CA, USA), fab161A conjugated with APC (R&D Systems, Minneapolis, USA), HLA-DR- (L243) specific mouse antibodies conjugated with pacific blue (biolegend, San Diego, CA, USA), MAC387 (Abcam, Cambridge UK), PE-conjugated CD11c (S-HCL-3) mouse antibodies (Becton Dickinson, New Jersey, USA), PE-conjugated mouse antibody CD83 (HB15A; Beckman Coulter, Miami Florida, USA), PE-conjugated mouse antibody CD163 (GHI/61; ebioscience, San Diego, CA, USA), CD150 antibody (A12; ABD Serotec, Dusseldorf, Germany), Alexa488- or Alexa647-labeled anti-mouse antibodies (Molecular probes, Eugene, OR, USA). MV infection was detected via EGFP in the FITC channel of the flow cytometer.

### Cell Lines and Viruses

Stable CHO transfectants expressing rhesus macaque DC-SIGN [Genbank AF391086] were generated using pRc/CMV-rh-DC-SIGN as previously described [17]. CHO cells expressing macaque DC-SIGN were selected with neomycin (1 mg/ml). CHO, Raji and Epstein-Barr virus-transformed B-lymphoblastic cell line (BLCL-GR) were cultured in RPMI1640 supplemented with L-glutamine (2 mM), penicillin (100 U/ml), streptomycin (100 mg/ml) and 10% (v/v) FCS. Generation of rMV^KS^EGFP for animal studies is described by Lemon *et al*. [20]. For *ex vivo* infection and transmission assays, rMV^KS^EGFP and rMV^IC323^EGFP were propagated on B-LCL cells or Vero-CD150 cells and titrated on Vero-CD150 cells for titer determination.

### FACS Analysis and Sorting

Flow cytometry was performed on a FACS Canto II or FACScan (BD Bioscience San Jose, CA, USA). Cell sorting was performed on a BD FacsAria. For all experiments, cells were washed in PBS/0.5% BSA and incubated with directly-labeled antibodies (1–5 µg/ml) or isotype controls (BD, San Jose, CA) for 30 minutes at 4°C. Samples were fixed with 4% (w/v) paraformaldehyde before measuring. Samples were analyzed by Cell Quest Pro software (BD Biosciences, San Jose, CA, USA) and experiments were analyzed using FlowJo 7.6.3 software (Tree star inc.).

### Immunohistochemical and Indirect Immunofluorescence Analysis

Formalin-fixed tissues were processed to paraffin. Consecutive sections (7 µm) from lung and lymphoid tissues were cut. Immunocytochemical staining was performed using a BondMax immunostainer with a polymer-based peroxidase detection system. DC-SIGN+ cells were detected using a monoclonal antibody to DC-SIGN (Invitrogen, ER2 antigen retrieval). Dual labeling indirect immunofluorescence was performed using monoclonal mouse antibodies macrophage marker MAC387 (Abcam, Cambridge UK) and DC-SIGN Mab161 (R&D Systems, Minneapolis, USA). Mab161 and MAC387 antibodies were visualized with a mixture of anti IgG1 and IgG2b conjugated with Alexa 546 or Alexa 488 (Molecular probes, Eugene, OR, USA). Nuclei were counterstained with Hoechst (Molecular probes, Eugene, OR, USA). All fluorescent stained slides were assessed with a Leica DMRA microscope and processed using Image Pro Plus software (Media Cybernatics). Matched isotype antibodies (BD, San Jose, CA, USA) served as negative control and were essentially blank.

### MV Infection and transmission Assays

For infection and transmission assays cells (10–50×10^3^) were seeded in a V-bottom plate and pre-incubated with mannan (0.25 mg/ml; Sigma-Aldrich, Zwijndrecht, Netherlands), anti-DC-SIGN (AZN-D1 or AZN-D2; 20 µg/ml) or IgG1 isotype control (20 µg/ml; BD, San Jose, CA, USA) for 30 minutes at 37°C, before incubation with rMV^IC323^EGFP or rMV^KS^EGFP at a multiplicity of infection (MOI) of 1–3 at 37°C for 24 hours in the case of infection. For transmission, after 2 hours the cells were washed and Raji (5×10^4^ cells) were added. After one to three days cells were harvested, washed and fixed with 4% (w/v) paraformaldehyde and EGFP expression was measured by flow cytometry. The gate for both populations was set at the uninfected control sample.

### Fluorescent Bead Adhesion Assay

The fluorescent bead adhesion assay was performed as described before [17]. In short, streptavidin was covalently coupled to carboxylate-modified TransFluorSpheres (488/645 nm by 1.0 µm; Molecular Probes, Eugene, OR, USA). The streptavidin-coated beads were incubated with biotinylated-sugars (Glycotech, Gaithersburg, MD, USA) at 4°C or biotinylated Goat-anti-human Fc (Jackson Immunoresearch, Baltimore, PA, USA) at 37°C and subsequently gp120 Fc at 4°C. The coated beads were added to cells at a ratio of 20∶1. Cells (10–20×10^3^) were incubated with beads for 45 minutes at 37°C. Mannan (0.25 mg/ml; Sigma-Aldrich, Zwijndrecht, Netherlands) and blocking antibodies against DC-SIGN (20 µg/ml) were used to determine the specificity of the adhesion. Binding was measured by flow cytometry.

### FITC-labeled MV Binding Assay

To generate FITC-labeled viruses, purified rMV^IC323^EGFP (2.4×10^7^ TCID50) was inactivated by dialyzing against 0.1% (w/v) formalin for 72 hours at 4°C. Next, viruses were labeled with FITC (0.1 mg/ml in 0.5 M bicarbonate buffer (pH 9.5) for 1 hour with constant stirring. The virus preparations were dialyzed against PBS overnight to remove unbound FITC. CHO cells (20×10^3^) were plated in 96 well v-bottom plates and pre-incubated with medium (RPMI 1640 medium, supplemented with L-glutamine, 10% (v/v) FCS, penicillin and streptomycin), mannan (0.25 mg/ml; Sigma-Aldrich, Zwijndrecht, Netherlands), blocking antibodies against DC-SIGN (AZN-D1) or IgG1 isotype control (BD, San Jose, CA) for 30 minutes at 37°C. Subsequently the cells were incubated with 20 µl of virus preparation for 30 minutes at 37°C. Binding was measured by flow cytometry.

### Fluorescently-labeled MV Binding Assay

MV labeling was performed as described by Hadac *et al* [43]. In short, MV-FSL-FLRO4 virions were prepared by adding 10 µl of FSL-FLRO4 (Kode Biotech, Auckland, New Zealand) (100 µg/ml) to 100 µl of rMV^KS^EGFP (2.2×10^6^ TCID50) or 100 µl RPMI (control), followed by incubation for 2 hours at 37°C. 20 µl of the prepared MV-FSL-FLRO4 or FLRO4-medium control was added to sorted cells (20×10^3^) in RPMI medium and incubated at 37°C for 30 minutes. Mannan (0.25 mg/ml; Sigma-Aldrich, Zwijndrecht, Netherlands) was used to determine the specificity of the binding. Following incubation the cells were washed and then fixed in 4% (w/v) paraformaldehyde before flow cytometry.

### Statistical Analysis

To evaluate differences between groups, a t test was used. P<0.05 was considered significant.
